# Variation in Butterfly Larval Acoustics as a Strategy to Infiltrate and Exploit Host Ant Colony Resources

**DOI:** 10.1371/journal.pone.0094341

**Published:** 2014-04-09

**Authors:** Marco Sala, Luca Pietro Casacci, Emilio Balletto, Simona Bonelli, Francesca Barbero

**Affiliations:** University of Turin, Department of Life Sciences and Systems Biology, Turin, Italy; University of Arizona, United States of America

## Abstract

About 10,000 arthropods live as ants' social parasites and have evolved a number of mechanisms allowing them to penetrate and survive inside the ant nests. Many of them can intercept and manipulate their host communication systems. This is particularly important for butterflies of the genus *Maculinea*, which spend the majority of their lifecycle inside *Myrmica* ant nests. Once in the colony, caterpillars of *Maculinea* “predatory species” directly feed on the ant larvae, while those of “cuckoo species” are fed primarily by attendance workers, by trophallaxis. It has been shown that *Maculinea* cuckoo larvae are able to reach a higher social status within the colony's hierarchy by mimicking the acoustic signals of their host queen ants. In this research we tested if, when and how myrmecophilous butterflies may change sound emissions depending on their integration level and on stages of their life cycle. We studied how a *Maculinea* predatory species (*M. teleius*) can acoustically interact with their host ants and highlighted differences with respect to a cuckoo species (*M. alcon*). We recorded sounds emitted by *Maculinea* larvae as well as by their *Myrmica* hosts, and performed playback experiments to assess the parasites' capacity to interfere with the host acoustic communication system. We found that, although varying between and within butterfly species, the larval acoustic emissions are more similar to queens' than to workers' stridulations. Nevertheless playback experiments showed that ant workers responded most strongly to the sounds emitted by the integrated (i.e. post-adoption) larvae of the cuckoo species, as well as by those of predatory species recorded before any contact with the host ants (i.e. in pre-adoption), thereby revealing the role of acoustic signals both in parasite integration and in adoption rituals. We discuss our findings in the broader context of parasite adaptations, comparing effects of acoustical and chemical mimicry.

## Introduction

Ants dominate most terrestrial ecosystems [1] and their colonies are so aggressively defended that they may act as shelters for any similar-sized organisms having evolved the necessary strategies to penetrate and live in their nests [2]–[5]. Myrmecophilous arthropods show various degrees of association with ants and spend variable proportions of their lives within or in the surroundings of ant colonies [1], [6]–[7]. The closer the relationship, the more specialised should be the “adaptations” needed to overcome colony barriers, break the communication codes of their hosts and become accepted as “self” by the workers' caste [2]–[5], [7]. Ants have evolved a complex set of signals which allow colony members to distinguish between nest-mates and intruders. Signals are mainly based on the exchange of chemical cues [1], [8]–[9] but also involve acoustic emissions [10]–[13]. Even though sound production is not usually the dominant strategy, acoustic communication plays a wide range of roles in the ants' social behaviour, from reciprocal attraction to inter-caste interactions and it has also been suggested that sounds are involved in the modulation of other signals (such as visual and chemical – e.g. [1]). In most cases, sound stimuli are effective only at small distances and are mainly used by ants for forager recruitment, mating requests, intimidation, aposematic “threatening”, or as signals of alarm [1], [14]–[16] (see [17] for a review of intracolony vibroacoustic communication).

A well-studied system in which parasites are known to co-opt both the chemical and the acoustical communication channels of their host ant is represented by *Maculinea* butterflies [18]. These lycaenids are obligate social parasites of *Myrmica* ants and have evolved several adaptations (e.g. behavioural, morphological, chemical and acoustic) for mimicking the honest signals of their host ants and using them to their own advantage [19].

*Maculinea* species show complex biological cycles and depend on specific host ants for their survival. Adults fly in early summer and females lay their eggs on species-specific host plants. Larvae feed inside flowers until their 4^th^ instar and finally drop to the ground. This part of the life cycle is the so-called “pre-adoption” phase. After that, they are “adopted” by *Myrmica* ants, which take the caterpillars into the brood chambers of their nests, starting the “post-adoption” phase of the butterfly's life cycle [18], [20]–[23]. In such a protected environment, the parasite will spend the next 11–23 months [24]. Within the ant nest, larvae lead a parasitic lifestyle and develop according to two different feeding strategies [21]. Larvae of *M. teleius* and *M. arion* actively feed on the ants' brood and are defined as predatory species. Larvae of *M. alcon* and *M. rebeli* are fed directly by the worker ants (trophallaxis) and are known as cuckoo feeders [21], [25]–[26]. Finally, the alimentary strategy of *M. nausithous* has not yet been fully clarified, with some authors suggesting the coexistence of both cuckoo and predatory strategies and others considering it as a cuckoo species (e.g. [27]–[28]).

After adoption, larvae of the predatory species spend much of their lives hidden in some remote chambers of the nest and contact with the host ants occurs only during their raids for preying on the brood. On the contrary, larvae of the cuckoo species become perfectly integrated members of the colony and compete with the ants' brood for the same resources [21].

Various authors [23], [29]–[30] have found evidence that chemical mimicry is used by *Maculinea* cuckoo species to bypass their host ants' recognition system. For predatory species only post-adoption data are available [31] showing that chemical mimicry is less specific than for cuckoo species. Only recently, the first case of acoustical mimicry in an ant social parasite has been demonstrated in the *Maculinea rebeli/Myrmica schencki* system [12] following a pilot study by DeVries et al. [32]. In detail, *Maculinea rebeli* (cuckoo species) larvae and pupae are able to mimic the sounds produced by *Myrmica schencki* queens, thus obtaining a high status in the host colony hierarchy [12], [33]–[34]. The ability to produce sounds similar to those emitted by *Myrmica sabuleti* queens was also shown for a *Maculinea* predatory species, *M. arion*, but the meaning and function of these acoustic emissions has not yet been assessed [35].

In this paper we investigate if acoustical mimicry can be related to the level of interaction between host and parasite. Specifically, we test if a *Maculinea* predatory species possesses butterfly-ant acoustic communication mechanisms, and compare results with those obtained from a cuckoo species. We evaluate if acoustic mimicry is instrumental for the parasite full integration into the ant colony by recording sound during both the pre-adoption and the post-adoption larval phase. We try to shed light on the functions of stridulations also as a possible mean for enhancing the adoption rituals. To this purpose, we compare two co-occurring populations of social parasites, *M. alcon* and *M. teleius* (respectively a cuckoo feeder and a predatory species), which exploit the same host ant species (*Myrmica scabrinodis*).

## Materials and Methods

### Ethics Statement

*Maculinea* caterpillars were collected under permit from The Italian Ministry for the Environment (protocol number: 446/05. DPN/2D/2005/13993). This permit covered all field studies.

### Study area

*Maculinea* larvae and worker ants were collected at Caselette (45°070N; 07°290E), about 15 km north-west of Turin, in northern Italy. Data collection took place within the Site of Communitarian Importance “Monte Musinè-Laghi di Caselette” (IT1110081) in a 2.9-ha wet grassland dominated by *Molinia coerulea*. The site is inhabited by three social parasites of *Myrmica* ants, i.e. *Maculinea alcon*, *Maculinea teleius* and *Microdon myrmicae*, all of which exploit only one *Myrmica* species, *M. scabrinodis* [36]. Adults of the two *Maculinea* butterflies overlap in time and space and their initial food plants, respectively *Gentiana pneumonanthe* and *Sanguisorba officinalis*, grow largely in the same meadow, so that larvae of the two species could parasitize the same colony [31]. This co-occurrence represents a key factor in the regulation of the population dynamics of the two species [37].

### Collection and sample maintenance

In June and July 2010 nine *Myrmica scabrinodis* nests were excavated in the field. At Caselette, *M. scabrinodis* colonies contain on average 200–500 workers, as well as from one to ten functional queens. In the laboratory we set up ant colonies of >100 workers in 28 cm×15 cm×10 cm Perspex containers and reared them on a diet of sugar and *Drosophila* larvae. To obtain pre-adoption larvae, at the end of the flight period of the two butterfly species (early September 2010) we gathered *Gentiana pneumonanthe* stalks with visible *M. alcon* eggs, as well as *Sanguisorba officinalis* plants, used by *M. teleius* caterpillars. As soon as larvae left their food plants as 4^th^ instars, they were recorded. We field collected the post-adoption larvae of the two parasite species and we kept them with their original *M. scabrinodis* host colonies. After 48 h of settlement in laboratory conditions, acoustic emission of caterpillar and ant samples were recorded. At the end of the experiments parasite larvae and ant samples were carried back to their original location.

### Sound recording and analysis

Recordings were made of individual workers (N = 11) and queens (N = 6) of *Myrmica scabrinodis*. We also recorded 15 pre-adoption larvae of *Maculinea alcon* and 5 of *M. teleius*, as well as 6 post-adoption *M. alcon* and 5 *M. teleius* caterpillars.

Samples were recorded for 20 minute periods, starting 10 min after an individual was introduced to the recording chambers and had become calm. During recording sessions, the specimens were placed on the microphone. Recordings were collected with custom recording equipment consisting of a 12.5 cm×8 cm×2 cm recording chamber with a moving-coil miniature microphone attached through the centre (sampling rate set to 44.10 kHz). A second microphone was used to record in anti-phase the ambient noise. The microphone output signal was processed through a two-stage low-noise amplification using a SP-24 B stereo microphone preamplifier (gain 53 dB).

Segments containing acoustic recordings were digitally saved in WAV format (16-bit amplitude resolution) on a laptop computer using Audacity v. 1.2.4 (http://audacity.sourceforge.net/). The equipment was powered by a 12V gel cell battery, and the recording chamber and microphones were located inside an anechoic chamber to further reduce ambient noise and interference [12].

For each file, the waveform and FFT spectrogram (FFT size = 512; Hanning window shape) were generated using Raven pro 1.3.

We measured 12 sound parameters for each pulse (Table S1, S2). On the 12 acoustic parameters we then computed a pairwise correlation analysis (Spearman-Rank-Correlation; Systat 8.0). From a pair of parameters with *r_s_*>0.75, only one was selected for multivariate analysis. Parameter pairs with *r_s_* <0.75 were defined as sufficiently non related [38]. This method yielded 4 acoustic variables: peak power (dB), peak frequency (Hz), IQRBW and pulse length (s), for which means±SD were calculated for further analysis.

A non-parametric One-Sample Kolmogorov-Smirnov Test was used to assess data distribution type.

We used Kruskal-Wallis tests with pairwise post hoc comparisons to verify whether the acoustic parameters differed between samples, and Principal Components Analysis (PCA) on the correlation matrix to describe patterns of variation in sound emissions [39]. ANOVA was performed to test differences between groups using the scores of the first two principal components. To further test whether the overall sounds differed between groups, we calculated the pairwise normalised Euclidean distances over all four parameters using the software Primer v6 (Primer-E Ltd.) and computed Student's t-test to estimate the significance of the differences. Data were analysed with SPSS20 package.

### Behavioural experiments

**Figure 1:**
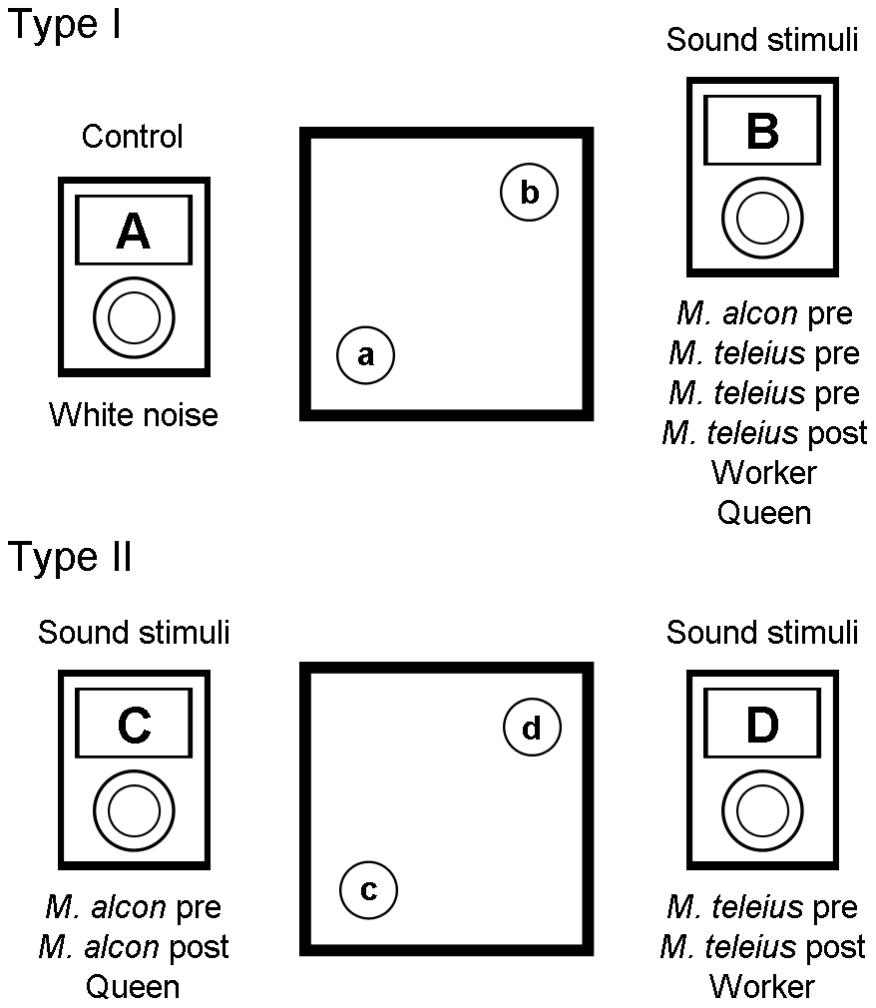
The two playback experimental setups. In the first set of playback bioassays (Type 1) the sounds emitted by *Myrmica scabrinodis* queens and workers and pre- and post-adoption *Maculinea alcon* and *M. teleius* larvae (stimuli) were tested against a white noise (control). In the second experimental setup (Type 2) we tested simultaneously two acoustic stimuli: the sounds of pre-adoption larvae of the two parasite species (*M. alcon* pre *vs*. *M. teleius* pre), the acoustic emissions produced by integrated parasite larvae (*M. alcon* post- *vs*. *M. teleius* post-adoption) and the sound of *M. scabrinodis* castes (Queen *vs*. Worker).

Two kinds of behavioural assays were carried out in 7 cm×7 cm×5 cm Perspex arenas with two speakers attached at the bottom of the box and covered with a thin layer of soil (Fig. 1).

Ten ant workers from the same colony were located in each arena and allowed to settle down for 20 minutes before the test sounds were played. Sounds were produced by two MP3 players playing loops of the original recordings. The volumes were adjusted to the natural level by connecting the speaker to the microphone of the recording equipment and calibrating to the same levels reached during the recording events. Each trial lasted 10 minutes and we recorded the number of times a selected behaviour was observed.

As in a previous experiment [13], we described 5 benevolent behaviours: *walking* – the ant worker walks towards and on the speaker without resting on it; *antennating* – the worker antennates the speaker for at least 5 seconds; *guarding* – the worker rests on the speaker in on-guard pose for at least 5 seconds; *alerting* – the worker abruptly changes direction to pass onto the speaker; *digging* – the worker digs the soil above the speaker.

In the Type 1 experimental setup each sound stimulus (i.e. sounds produced by queen ants, worker ants, pre- and post-adoption *M. alcon* larvae, pre- and post-adoption *M. teleius* larvae) was tested against white noise (control) for each of 9 *Myrmica* colonies (Fig. 1).

In the second set of bioassays (Type 2) we performed cross tests by comparing the behaviours elicited by two sound stimuli played contemporaneously (Fig. 1). We played the acoustic emissions produced by pre-adoption larvae of *M. alcon vs. M. teleius*, post-adoption larvae of *M. alcon vs. M. teleius*, ant workers *vs.* queens.

The frequencies of behavioural responses to the sound stimuli were analysed using Chi Square tests.

## Results

### Sound recordings

**Figure 2:**
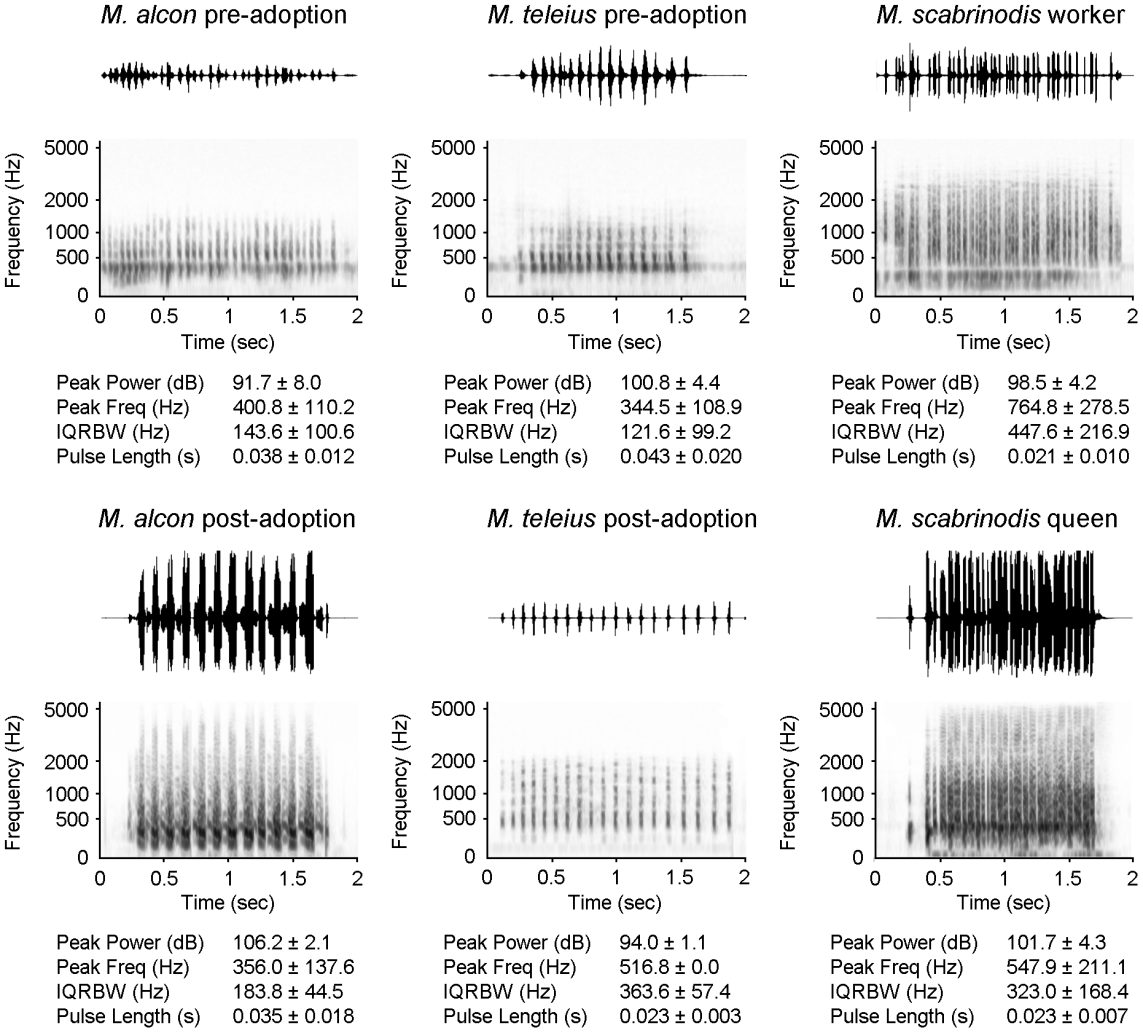
Sound emissions of *Maculinea* larvae and *Myrmica* ants. Example waveforms (upper traces) and spectrograms (lower traces) of sounds emitted by pre-adoption and post-adoption larvae of the two parasites (*Maculinea alcon* and *M. teleius*) and stridulations produced by *Myrmica scabrinodis* queens and workers. Mean ± SD of the four pulse parameters are also reported for each insect category.

We recorded and analysed acoustic emissions from pre- and post-adoption caterpillars of *M. alcon* (Audio S1, S2) and *M. teleius* (Audio S3, S4), as well as from workers (Audio S5) and queens (Audio S6) of *Myrmica scabrinodis*, for a total of 429 pulses. Average measurements for the four sound parameters are listed in Figure 2.

*Maculinea* stridulations consist of a series of pulse trains (series of close “clicks”), each lasting about 2 seconds and are on average made of 20 pulses (clicks). Ants' pulse trains are slightly more durable, lasting about 5 seconds and are on average formed by 40 pulses (Fig. 2).

Our data show that sounds produced by queens and workers of *Myrmica scabrinodis* are distinctive on the basis of their intensity (peak power) and peak frequency (Fig. 2; Table S3). The pulses produced by queens are emitted at a higher intensity than those of workers and are characterised by lower frequencies (Fig. 2; Tables S2, S3). The pre-adoption larvae of both butterfly species produced sounds distinguishable from those emitted during the post-adoption phase. The main differences concerned sound intensity, which in *M. alcon* is higher for post-adoption than for pre-adoption larvae, while in *M. teleius* larvae it decreases, in contrast, from pre- to post-adoption (Fig. 2; Table S2, S3). Both the predatory and the cuckoo species showed a significant increase in the IQRBW (linked to frequency - see Fig. 2; Table S2, S3), passing from pre- to post-adoption phases.

When we compared single components of the sounds emitted by *M. alcon* larvae in the post-adoption phase with those of ants, we found no differences from queens' stridulations and significant dissimilarities from those of workers. The calls produced by post-adoption *M. teleius* larvae (inside the nest), in contrast, were similar to those of workers and distinguishable from those of queen by one intensity-linked parameter (peak power) (Fig. 2; Table S3).

**Figure 3:**
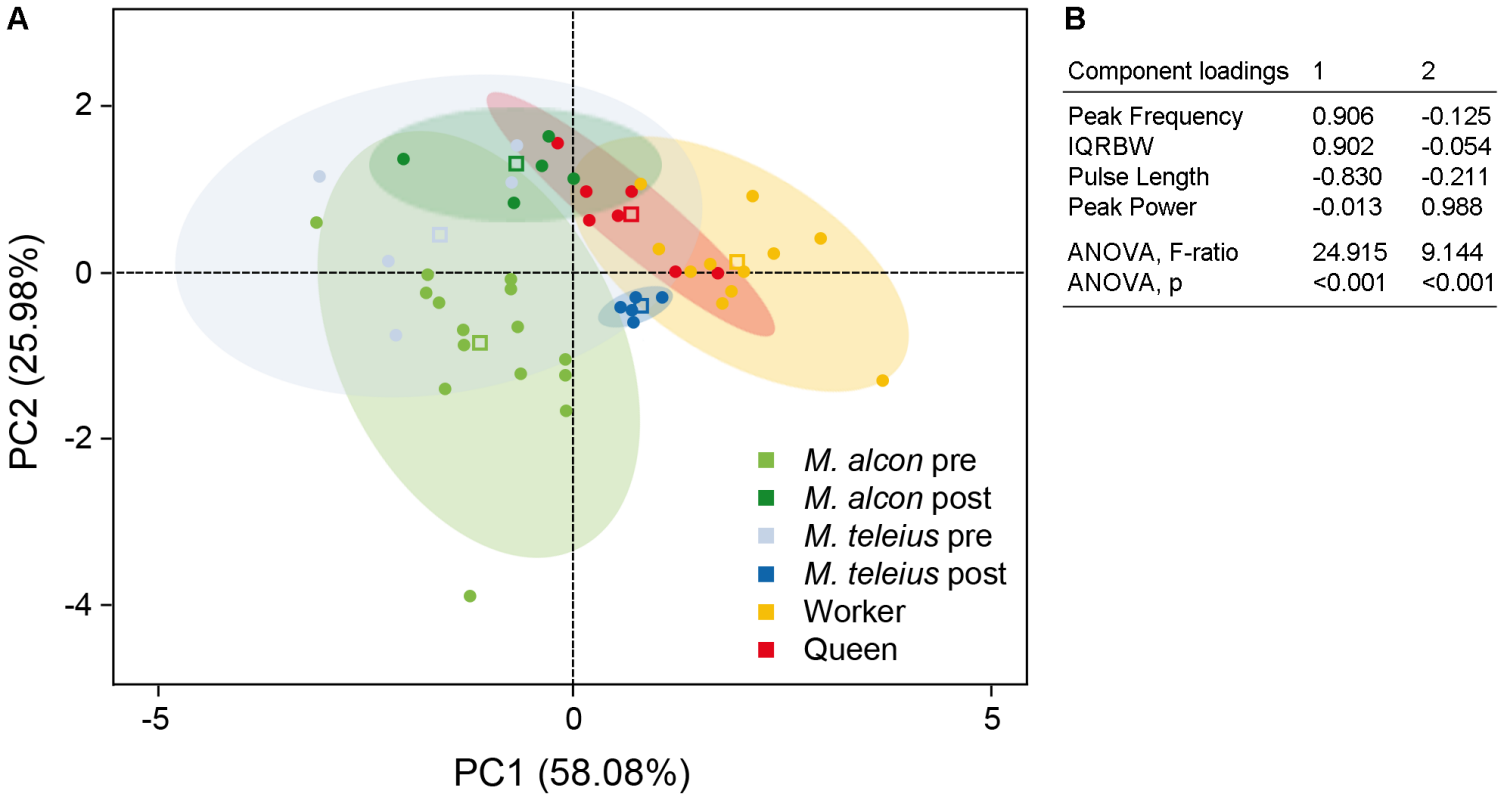
Principal Components Analysis (PCA) on the four sound parameters. (a) Two-dimensional plot of the first two factors extracted by principal components analysis over all individual measurements of the four sound parameters (peak frequency, peak power, IQRBW and pulse length) for each insect category - *Myrmica scabrinodis* queens and workers and *Maculinea alcon* and *M. teleius* pre- and post-adoption caterpillars. Ellipses indicate 95% confidence intervals; squares show the centroids for each category. (b) The component loadings extracted by PCA from the four sound parameters are reported in the table. ANOVA based on the 6 groups of samples are also reported.

Principal Component Analysis, carried out on the four sound parameters recorded from specimens of *Maculinea* larvae and *Myrmica* ant castes (21 *M. alcon* and 10 *M. teleius* larvae, 11 workers and 6 queens from different *M. scabrinodis* nests) generated two components with eigenvalues greater than 1, which significantly discriminated between groups (Fig. 3).

The first component explained 58% of total variance and was influenced by two spectrum parameters (peak frequency, IQRBW) and by pulse length. The second component accounted for 26% of total variance and significantly discriminated between groups on the basis of peak power.

As showed in the two-dimensional plot of the two factors extracted by principal components analysis, the first component clearly separated *Maculinea* butterflies' larvae from *Myrmica* ant castes with the exception of *M. teleius* post-adoption larvae. The second component separated pre- and post-adoption phase of both butterfly species.

Normalised Euclidean distances (mean ± SD) between butterfly instars and the two ant castes are reported in Table 1.

**Table 1:** Average Euclidean distances between sounds emitted by parasite larvae and ants.

	Queens	Workers
*M. alcon* pre-adoption larvae	2.515±0.567	3.387±0.846
*M. alcon* post-adoption larvae	1.464±0.476	2.918±0.703
*M. teleius* pre-adoption larvae	2.637±1.179	3.804±1.193
*M. teleius* post-adoption larvae	1.589±0.507	1.867±0.643

Normalised Euclidean distances (mean ± SD) between the sounds produced by larvae of the two parasite species (*Maculinea alcon* and *M. teleius*) in pre-adoption and post-adoption phases and the stridulations emitted by *Myrmica scabrinodis* queens and workers. Euclidean distances were calculated using the four sounds parameters: peak power (dB), peak frequency (Hz), IQRBW (Hz), pulse length (s).

The signals emitted by butterfly larvae in pre- and post-adoption were significantly closer to the stridulations of queens than to those of workers (two-sample t test: t *_M. alcon_*_ pre-adoption_ = −8.854, df = 191, p<0.001; t *_M. alcon_*_ post-adoption_ = −8.327, df = 58, p<0.001; t *_M. teleius_*_ pre-adoption_ = −4.011, df = 73, p<0.001) with the only exception of *M. teleius* post-adoption larvae (two-sample t test: t *_M. teleius_*_ post-adoption_ = −1.885, df = 58, p = 0.063). For each of the butterfly species, the sounds emitted in the post-adoption phase resembled those of the queens more than those produced in pre-adoption (two-sample t test: t *_M. alcon_* = 7.549, df = 88, p<0.001; t *_M. teleius_* = 4.083, df = 48, p<0.001). In both butterfly species sounds differed between the pre- and the post-adoption phase and this change was significantly more obvious in the predatory species (*M. alcon*_pre-post-adoption_ = 2.319±0.560; *M. teleius*_pre-post-adoption_ = 2.956±1.019; t test = −2.966, df = 93, p<0.001).

Interestingly, the sound similarity between both *M. alcon* and *M. teleius* post-adoption larvae and queen ants was higher than between stridulations emitted by queens and their workers' (two-sample t test: t *_M. alcon_* = −4,020, df = 54, p<0.001; t *_M. teleius_* = −3,267, df = 66, p = 0.002).

### Behavioural experiments

For playback experiments, nine colonies of *Myrmica scabrinodis* were used, for a total of 81 playbacks and 13.5 hours of observation. No antagonistic or alarmed behaviours were observed.

**Figure 4:**
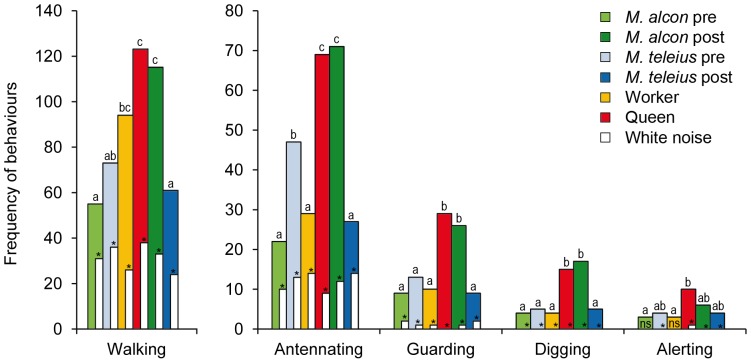
Worker ant reactions to sound stimuli and white noise. When the sound stimuli (color bars) were played simultaneously against the white noise (white bars), they always elicited higher behavioural responses on worker ants (experimental setup Type 1). Comparing the sound stimuli, we found that stridulations produced by queens caused stronger reactions in *M. scabrinodis* ants than those emitted by workers (red *vs*. yellow bars). Workers reacted more frequently to sounds produced by *M. alcon* integrated larvae than those emitted by *M. alcon* pre-adoption caterpillars (dark green *vs*. pale green) while on the contrary, the sounds emitted by *M. teleius* pre-adoption larvae caused more frequent reactions in workers than those produced by post-adoption larvae (light blue *vs*. dark blue) significantly for “antennating”. Different letters indicate significantly different behavioural responses elicited by sound stimuli (Chi square Yates' correction). Significantly (p<0.05) different behavioural frequencies between white noises and sound stimuli are indicated by asterisks. ns  =  statistically not significant.

During playback experiments (Type 1), the acoustic stimulus always elicited higher benevolent behavioural responses in workers of *M. scabrinodis* (for all the five behaviours observed) than the white noise, used as control (Fig. 4). If we consider counts of each behaviour elicited by sound stimuli, “walking” represented 54%, “antennating” accounted for 27%, “guarding” for 9%, “digging” for 5% and “alerting” for 3% of total workers' responses.

The stimuli which most clearly increased worker ants' attention were sounds emitted by queen ants and by the cuckoo species in the post-adoption phase (responses to these two stimuli were not statistically different; Fig. 4).

Using cross tests to compare reactions elicited in worker ants by the two butterfly species in the pre-adoption phase of their life cycle (Experiment Type 2), we observed that sound produced by the predatory species promoted slightly stronger responses from the host ants than that of cuckoo species (χ^2^ = 4.033, df = 1, p = 0.045) significantly as concern the “antennating” behaviour (χ^2^ = 6.425, df = 1, p = 0.011). In the post-adoption phase (Experiment Type 2), sounds produced by the cuckoo species elicited a significantly stronger response from the host ants (χ^2^_Tot_ = 24.235, df = 1, p<0.001), specifically in “walking” (χ^2^ = 8.195, df = 1, p = 0.004), “antennating” (χ^2^ = 10.257, df = 1, p = 0.001) and “digging” (χ^2^ = 5.556, df = 1, p = 0.018). When ants received simultaneously stridulations produced by queens and workers (Experiment Type 2) reacted more frequently to the queen sounds (χ^2^_Tot_ = 9.531, df = 1, p = 0.002).

## Discussion

Communication is fundamental for social insects, such as ants, which live in complex hierarchical societies and need to function collectively as a “super-organism” [8].

Although ant communication primarily relies on the exchange of chemical cues [1], sounds are also emitted by ants in several circumstances, both outside (e.g. [40]–[41]) and inside their colonies ([17] and references therein). By studying *Myrmica schencki*, we recently discovered that ant colony members are capable of producing caste-specific acoustic emissions, which all have the potential to influence the behaviour of equal-rank ants, as well as other castes [12]. Along with *M. scabrinodis* queens and workers, sclerotised pupae also emit sounds to communicate with nurse workers [13]. In previous articles [12], [35], we have described differences between the stridulations emitted by queens and workers of both *M. schencki* and *M. scabrinodis* ants, according to three parameters: *pulse length*, *pulse repetition frequency* and especially *peak frequency*. Here, we have carried out further investigations into the stridulations of *M. scabrinodis* by analysing some additional sound parameters, and we have confirmed that workers' and queens' stridulations are markedly different (Fig. 2; Table S2, S3). In addition, we assayed the functions of these sounds in playback experiments, and our data revealed that stridulations made by *M. scabrinodis* queens resulted in more obvious reactions from the ants compared to stridulations produced by their own workers, for almost all observed behaviours (Fig. 4; Experiment Type 2). The analysis of ant stridulations has indicated that acoustic signalling is often used in ant-ant communication, and could act as a barrier for any myrmecophilous organism wishing to enter and exploit a colony. Some intruders, however, are able to employ acoustic mimicry to overcome this barrier, and thus break the host ants' communication code. During playback bioassays, *Myrmica scabrinodis* workers reacted with non-aggressive behaviours in response to sounds produced by intruding *Maculinea* parasitic butterfly larvae.

### Comparisons between acoustic emissions of predators and cuckoo species in the post- adoption phase of their lifecycle

In this study, we demonstrated the ability of two *Maculinea* species (a cuckoo species and a predatory species) to break their host's communication code by mimicking the acoustic signals of the host ants (Fig. 4). Our data have demonstrated that, in the post-adoption phase, stridulations emitted by the two species of *Maculinea* are distinguishable from each other, but appear to be equally similar to those of the ant queens (Table 1). It is worth noting that the overlap between the acoustic emissions of the two parasites with those produced by the queens is linked to different sound features (Fig. 3, see below for details). Currently, however, we do not know how particular sounds are perceived by the ants [42]–[43], and whether a single sound component may be more informative than others. Nevertheless, our playback experiments have allowed us to assess the overall effect of acoustic emissions in the ant colony, and which sound stimulus elicits the highest response in workers. Here, we have demonstrated that, despite both parasite species producing similar sounds to those of *M. scabrinodis* queens, the sounds produced in the post-adoption phase by the cuckoo species (*Maculinea alcon*) tended to promote a significantly higher number of reactions by the workers compared to sounds produced by *M. teleius*. Workers mainly reacted to the cuckoo species sounds by “walking”, thereby suggesting an attraction towards the sound source, and by “antennating”, a behaviour employed in contexts such as food exchange, recruitment and nest-mate recognition [44]–[46] (Fig. 4; Experiment Type 2). These induced behaviours are consistent with the needs of highly integrated *M. alcon* larvae, which are fed and nursed by ants by means of trophallaxis [26], [47]. Interestingly, sounds produced by *M. alcon*, similar to those of queen ants, more frequently elicit the “digging” behaviour, compared to sounds emitted by the predatory species, *M. teleius* (Fig. 4). It is well established that ants dig in order to find nest-mates trapped under the soil [48], [49], and in the case of *Atta* spp., for example, a role of stridulation in eliciting the rescue behaviour has been demonstrated [50]–[51]. Thus, the parasite sound stimulus alone was able to elicit a reaction in the host ants, consistent with the subsequent rescue behaviour. The rescue is promoted by cuckoo species during disturbance of the host nest and has been observed in laboratory experiments where cuckoo species larvae are retrieved by workers in preference to their own larvae [25].

According to results from our multivariate analysis, the emissions of *M. alcon* and *M. scabrinodis* queens overlap in the second principal component, which is mainly correlated to sound intensity. The cuckoo species emitted sounds that exceed in intensity by 4 dB, compared to those of the queens, while emissions of the predator parasite were 8 dB lower than those of the queens. It is known that Lepidoptera are able to distinguish between acoustical emissions that differ by about 1–2 dB [52]. Moreover, females of the wax moth *Achroia grisella* can distinguish between males on the basis of calls that have been artificially modified in just a single sound component, tending to favour acoustic emissions that are louder, delivered at higher rates, and with more evenly spaced pairs of pulses [52]. We therefore speculate that parameters linked to sound intensity (dB) could be among the most informative components of acoustic stimuli in the *Maculinea-Myrmica* system.

Despite the fact that sounds produced by *M. teleius* post-adoption larvae were characterised by lower intensities than those of the queens, playback experiments demonstrate that these particular sound stimuli are still able to induce reactions in ant workers (Fig. 4). The number of ant responses to sounds of post-adoption *Maculinea* predatory species was among the lowest recorded, for each behaviour, although significantly more ant reactions were induced by emissions from the predatory species compared to the control stimulus. This is consistent with a predatory life style; by preying directly on ant larvae, *M. teleius* larvae do not need to activate such an elaborate acoustical strategy as that of the cuckoo species, after they have been adopted into the ant colony [33].

### Comparisons between the acoustic emissions of predator and cuckoo species in the pre-adoption phase of their lifecycle

When *Maculinea* larvae abandoned their food plants, and are still outside the ant nest, the ability to produce sounds that solicit the attention of foraging ants could be a great advantage. Pre-adoption *Maculinea* larvae (both *M. alcon* and *M. teleius*) emitted sounds that are much more similar to those of ant queens than to those of ant workers (Table 1, S3). If intensity is considered as being one of the most informative features of the sound, it is worth noting that in its early (pre-adoption) life stages, *M. teleius* produce calls at an intensity that perfectly overlapped those emitted by queen ants, while pre-adoption *M. alcon* larvae emitted sounds at a lower intensity than the ants. This observation fully correlates with the findings that *M. scabrinodis* workers reacted showing a higher amount of responses to the emissions produced by the pre-adoption larvae of *M. teleius* (predator), compared to those of *M. alcon* (cuckoo) larvae (Fig. 4; Experiment Type 2).

According to previous studies [22]–[23], the quick retrieval of *Maculinea* larvae is supposedly mediated only by the chemical mimicry of surface hydrocarbons existing on the epicuticle of *Myrmica* workers. Cuckoo larvae are commonly retrieved in a few minutes, thanks to the synthesis of specific epicuticular hydrocarbons [29]–[30]. However, this chemical mechanism has only been assessed for cuckoo species, while the only evidence for predatory species is that the adoption ritual is more durable than that of cuckoo species [53]–[54]. If a predatory larva, such as *M. teleius*, is found by a *Myrmica* forager, it has to perform complex “adoption” behaviours (including secretions from the dorsal nectary organ), that could last for hours [54]. It has been proven that there is a proportionally shorter adoption time if the match between the surface chemistry of the *Maculinea* parasite and its *Myrmica* host is greater [30], which provides indirect evidence that the chemical mimicry employed by predator species might be less effective than that of cuckoo species. We suggest a previously undetected role for acoustic signals outside the nest in the adoption process. During the long adoption rituals, the predatory species (*M. teleius*) may use acoustical emission to complement its chemical mimicry, by increasing and maintaining the attention of forager ants required for recognition as a colony member. In playback bioassays, ant workers mainly responded to the sounds of *M. teleius* pre-adoption larvae by “antennating” the speaker (Fig. 4), a behaviour that is considered by many authors as a sensitive measure of the nest-mate discrimination ability of ants (e.g. [1], [55]).

### Comparisons between pre- and post-adoption phases

Our data reveal that acoustical signatures unexpectedly change in both *Maculinea* species from the pre- to the post-adoption phase. Once inside the nest, the intensity of *M. alcon* emissions increases, and becomes more similar to those of ant queens, compared to the pre-adoption phase, while sounds produced by the predatory larvae show an opposite trend. If changes were attributable to larval growth and increase in size, we would have expected similar acoustic patterns to occur in both parasite species. Currently, however, we are unable to relate acoustic differences to any particular kind of structural variation. Structures similar to ant stridulatory organs, formed of a plectrum and a file, are not present on the cuticles of *Maculinea* larvae. However, in the mature post-adoption larvae, we were able to observe a tiny tooth-and-comb organ [12], structurally similar to those described in the mutualistic Australian lycaenid *Arhopala madytus* [56]. In addition, Schurian et al. [57] suggested that stridulations can also be generated by compressing air, as a result of larval abdominal muscle contractions. It is certain, therefore, that distinct sound-producing structures do exist in butterfly parasites and host ants, and it follows that the different structures have been channeled, by evolution, into producing very similar stridulations. On the other hand, supposedly similar organs would be able to emit sounds distinct in the two butterfly species and in two separate moments of the lifecycle, accordingly to their needs in terms of interaction with host ants.

## Conclusions

In this study, we have reported a plasticity in usage and reception of acoustic signalling between butterfly social parasites and host ants. Here we provide, for the first time, evidence of a role for sounds in the pre-adoption period, when harmless *Maculinea* larvae are still outside the nest. In a similar manner to *M. rebeli* [12], we have demonstrated that *M. alcon* also uses sounds to achieve a high social status in the colony hierarchy, thus being treated as queen ants.

Both butterfly species, at each stage of their lifecycle, are in fact able to mimic the sounds produced by queen ants, but they are not able to elicit the same number of benevolent responses in ants. By means of playback experiments, we have been able to univocally demonstrate that acoustical patterns vary across the species, and according to the various degrees of interaction shown in the two topical moments of the butterfly life cycle (pre- and post-adoption phases). Due to the scarce knowledge of sound emission and/or reception mechanism in such a study system, any research about the role of acoustic communication should involve the use of adequate behavioural experiments to avoid partial or wrong conclusions.

The fact that *Maculinea* “acoustic strategies” vary according to life history traits, in relation to the species' feeding behaviour, and according to larval development, reveals that sounds can convey an effective message in various contexts, fitting the definition of biological communication [58], in which both the signal and response are adaptive.

## Supporting Information

Table S1 **Description of the twelve sound parameters according to the definitions provided by the Raven Pro 1.3 user's manual.**
(DOCX)

Table S2 **Mean ± SD for the twelve sound parameters collected for all the species groups.**
(DOCX)

Table S3 **Post hoc univariate pairwise comparisons of the four sound parameters between species groups.** pre  =  pre-adoption larvae; post  =  post-adoption larvae; ns  =  statistically not significant; * p<0.05; ** p<0.01.
(DOCX)

Audio S1 **Series of pulses from a** ***Maculinea alcon*** **pre-adoption larva.**
(WAV)

Audio S2 **Series of pulses from a** ***Maculinea alcon*** **post-adoption larva.**
(WAV)

Audio S3 **Series of pulses from a** ***Maculinea teleius*** **pre-adoption larva.**
(WAV)

Audio S4 **Series of pulses from a** ***Maculinea teleius*** **post-adoption larva.**
(WAV)

Audio S5 **Series of pulses from a** ***Myrmica scabrinodis*** **worker.**
(WAV)

Audio S6 **Series of pulses from a** ***Myrmica scabrinodis*** **queen.**
(WAV)
