# Supplementary material for: Variation in Butterfly Larval Acoustics as a Strategy to Infiltrate and Exploit Host Ant Colony Resources
Source: PLoS One. 2014 Apr 9;9(4):e94341. doi: 10.1371/journal.pone.0094341 (PMC3981827; doi:10.1371/journal.pone.0094341)
Supplement: Table S1 — Description of the twelve sound parameters according to the definitions provided by the Raven Pro 1.3 user's manual. (DOCX) [file pone.0094341.s001.docx]

**Table S1. Description of the twelve sound parameters according to the definitions provided by Raven Pro 1.3 user’s manual.**

| **Spectral parameters** | |
| --- | --- |
| ***Average Power*** | the value of the power spectrum averaged over the frequency extent of the selection. The values of the power spectrum are summed between the lower and upper frequency bounds of the selection, and the result is divided by the number of frequency bins in the selection. Unit: dB |
| ***Energy*** | the total energy within the selection bounds. For a spectrogram, the energy is calculated as:  $\left( \sum_{t=t_{1}}^{t_{2}} \sum_{f=f_{1}}^{f_{2}} \left( W_{0}\cdot{10}^{\left( S_{t,f}/10 \right)} \right) \right)\Delta f$  where *f_1_* and *f_2_* are the lower and upper frequency limits of the selection, t_1_ and t_2_ are the beginning and ending frame numbers of the selection, *W_0_* is the power dB reference value, *S_t,f_* is the spectrogram power spectral density in frame *t* at frequency *f* (in dB), and Δ*f* is the frequency bin size |
| ***Peak Power*** | the maximum power in the selection. In a gray scale spectrogram, the maximum power in a selection is the power at the darkest point in the selection. Unit: dB relative to 1 dimensionless sample unit. |
| ***1st Quartile Frequency*** | the frequency that divides the selection into two frequency intervals containing 25% and 75% of the energy in the selection. Unit: Hz |
| ***3rd Quartile Frequency*** | the frequency that divides the selection into two frequency intervals containing 75% and 25% of the energy in the selection. Unit: Hz |
| ***Peak Frequency*** | the frequency at which Peak Power occurs within the selection. Unit: Hz |
| ***IQR (Inter-quartile Range) Bandwidth*** | the difference between the 1st and 3rd Quartile Frequencies. Unit: Hz |
| **Waveform parameters** | |
| ***Max Amplitude*** | the maximum of all the sample values in the selection |
| ***Min Amplitude*** | the minimum of all the sample values in the selection |
| ***Peak Amplitude*** | the greater of the absolute values of Max Amplitude and Min Amplitude |
| ***RMS Amplitude*** | the root‐mean‐square amplitude (sometimes called “effective amplitude”) of the selected part of the signal. RMS amplitude is equal to:  $\sqrt{\sum_{i=1}^{n} \frac{x_{i}^{2}}{n}}$  where *n* is the number of samples in the selection, and *xi* is the amplitude (in dimensionless sample units) of the *i*_th_ sample in the selection. |
| ***Pulse length (Delta time)*** | the pulse duration. Unit: second |
