# Supplementary material for: Variation in Butterfly Larval Acoustics as a Strategy to Infiltrate and Exploit Host Ant Colony Resources
Source: PLoS One. 2014 Apr 9;9(4):e94341. doi: 10.1371/journal.pone.0094341 (PMC3981827; doi:10.1371/journal.pone.0094341)
Supplement: Table S2 — Mean ± SD for the twelve sound parameters collected for all the species groups. (DOCX) [file pone.0094341.s002.docx]

|  | *M. scabrinodis*  Workers | *M. scabrinodis*  Queens | Pre-adoption  *M. alcon* larvae | Pre-adoption  *M. teleius* larvae | Post-adoption  *M. alcon* larvae | Post-adoption  *M. teleius* larvae |
| --- | --- | --- | --- | --- | --- | --- |
| Number of pulses | 132 | 72 | 150 | 51 | 15 | 9 |
| Avg Power (dB) | 79.2 ± 3.7 | 81.2 ± 4.0 | 67.9 ± 7.4 | 76.4 ± 4.9 | 84.4 ± 2.7 | 70.8 ± 1.0 |
| Energy (dB) | 83.0 ± 4.2 | 85.7 ± 4.4 | 74.6 ± 7.8 | 83.3 ± 4.2 | 90.4 ± 4.0 | 75.6 ± 1.0 |
| Peak Power (dB) | 98.5 ± 4.2 | 101.7 ± 4.3 | 91.7 ± 8.0 | 100.9 ± 4.4 | 106.2 ± 2.1 | 94.0 ± 1.1 |
| Q1 Freq (Hz) | 614.7 ± 161.0 | 440.2 ± 115.3 | 351.4 ± 89.0 | 307.4 ± 86.5 | 321.5 ± 60.6 | 382.8 ± 76.0 |
| Q3 Freq (Hz) | 1062.3 ± 223.4 | 763.2 ± 163.1 | 495.0 ± 147.1 | 429.0 ± 159.3 | 505.3 ± 44.5 | 746.5 ± 86.1 |
| Peak Freq (Hz) | 764.8 ± 278.5 | 547.9 ± 211.1 | 400.8 ± 110.2 | 344.5 ± 108.9 | 356.0 ± 137.6 | 516.8 ± 0.0 |
| IQRBW (Hz) | 447.6 ± 216.9 | 323.0 ± 168.4 | 143.6 ± 100.6 | 121.6 ± 99.2 | 183.8 ± 44.5 | 363.6 ± 57.4 |
| Pulse length (s) | 0.021 ± 0.010 | 0.023 ± 0.007 | 0.038 ± 0.012 | 0.043 ± 0.020 | 0.035 ± 0.018 | 0.023 ± 0.003 |
| Max Amp | 17535 ± 6790 | 21749 ± 8140 | 6743 ± 4804 | 14164 ± 6137 | 27535 ± 4572 | 7423 ± 1100 |
| Min Amp | -15890 ± 5620 | -20187 ± 6568 | -6573 ± 4470 | -14468 ± 5372 | -23247 ± 3821 | -8293 ± 956 |
| Peak Amp | 18741 ± 6457 | 22978 ± 7901 | 7045 ± 4914 | 15160 ± 6165 | 27535 ± 4572 | 8348 ± 941 |

**Table S2. Mean ± SD for the twelve sound parameters collected for all the species groups.**
