# Supplementary material for: Variation in Butterfly Larval Acoustics as a Strategy to Infiltrate and Exploit Host Ant Colony Resources
Source: PLoS One. 2014 Apr 9;9(4):e94341. doi: 10.1371/journal.pone.0094341 (PMC3981827; doi:10.1371/journal.pone.0094341)
Supplement: Table S3 — Post hoc univariate pairwise comparisons of the four sound parameters between species groups. pre = pre-adoption larvae; post = post-adoption larvae; ns = statistically not significant; * p<0.05; ** p<0.01. (DOCX) [file pone.0094341.s003.docx]

|  |  |  | *M. alcon* | | *M. teleius* | | *M. scabrinodis* | |
| --- | --- | --- | --- | --- | --- | --- | --- | --- |
|  |  |  | pre | post | pre | post | Queens | Workers |
| *M. alcon* | pre | Peak Power (dB) | --- | 271.51 ** | 158.83 ** | ns | 178.59 ** | 110.17 ** |
|  |  | Peak Freq (Hz) | --- | ns | ns | ns | 81.04 ** | 163.33 ** |
|  |  | IQRBW (Hz) | --- | 117.26 * | ns | 203.95 ** | 176.72 ** | 219.80 ** |
|  |  | Pulse Length (s) | --- | ns | ns | ns | 125.95 ** | 171.91 ** |
|  | post | Peak Power (dB) |  | --- | 112.68 * | 274.83 ** | ns | 161.34 ** |
|  |  | Peak Freq (Hz) |  | --- | ns | ns | ns | 176.44 ** |
|  |  | IQRBW (Hz) |  | --- | 130.79 ** | ns | ns | 102.54 * |
|  |  | Pulse Length (s) |  | --- | ns | ns | ns | 127.37 * |
| *M. teleius* | pre | Peak Power (dB) |  |  | --- | 162.16 * | ns | ns |
|  |  | Peak Freq (Hz) |  |  | --- | ns | 125.57 ** | 207.85 ** |
|  |  | IQRBW (Hz) |  |  | --- | 217.48 ** | 190.25 ** | 233.33 ** |
|  |  | Pulse Length (s) |  |  | --- | ns | 128.11 ** | 174. 08 ** |
|  | post | Peak Power (dB) |  |  |  | --- | 181.91 ** | ns |
|  |  | Peak Freq (Hz) |  |  |  | --- | ns | ns |
|  |  | IQRBW (Hz) |  |  |  | --- | ns | ns |
|  |  | Pulse Length (s) |  |  |  | --- | ns | ns |
| *M. scabrinodis* | Queens | Peak Power (dB) |  |  |  |  | --- | 68.42 * |
|  |  | Peak Freq (Hz) |  |  |  |  | --- | 82.28 * |
|  |  | IQRBW (Hz) |  |  |  |  | --- | ns |
|  |  | Pulse Length (s) |  |  |  |  | --- | ns |
|  | Workers | Peak Power (dB) |  |  |  |  |  | --- |
|  |  | Peak Freq (Hz) |  |  |  |  |  | --- |
|  |  | IQRBW (Hz) |  |  |  |  |  | --- |
|  |  | Pulse Length (s) |  |  |  |  |  | --- |

**Table S3. Post hoc univariate pairwise comparison of the four sound parameters between species groups.** pre = pre-adoption larvae; post = post-adoption larvae; ns statistically not significant; * p<0.05; ** p<0.01.
